# Multiple socioeconomic circumstances and trajectories of fruit and vegetable consumption: the Helsinki Health Study

**DOI:** 10.1177/14034948221094430

**Published:** 2022-05-10

**Authors:** Elina Mauramo, Jatta Salmela, Leonie H. Bogl, Tea Lallukka, Noora Kanerva

**Affiliations:** 1 Department of Public Health, University of Helsinki, Helsinki, Finland; 2 Institute for Molecular Medicine Finland (FIMM), University of Helsinki, Helsinki, Finland; 3 Department of Epidemiology, Medical University of Vienna, Vienna, Austria

**Keywords:** Socioeconomic position, fruit and vegetable use, trajectories, employee cohort

## Abstract

**Aims:**

Fruit and vegetable consumption is essential in disease prevention. Socioeconomic differences in consumption have been observed but evidence from longitudinal studies incorporating multiple socioeconomic indicators is lacking. We examined long-term fruit and vegetable consumption trajectories and multiple socioeconomic circumstances as their determinants.

**Methods:**

We used survey data from the Helsinki Health Study (phase 1 in 2000–2002, *N*=8960, response rate 67%; phases 2–4 in 2007, 2012 and 2017) among initially 40- to 60-year-old employees of City of Helsinki, Finland. Fruit and vegetable consumption was measured by a food frequency questionnaire and consumption times per month were calculated. Childhood (parental education, economic difficulties), conventional (own education, occupational class, household income) and material (housing tenure, wealth, current economic difficulties) socioeconomic circumstances were included. We used group-based trajectory modelling for identifying fruit and vegetable consumption trajectories and multinomial logistic regression for examining associations between socioeconomic circumstances and the trajectories.

**Results:**

Four fruit and vegetable consumption trajectories were identified: increasing higher (12%), decreasing higher (10%), stable moderate (43%) and stable low (35% of participants). Childhood, conventional and material socioeconomic circumstances were all associated with the trajectories: belonging to a lower socioeconomic group was associated with belonging to the stable low and moderate trajectories. In multivariate models, the strongest associations were found for occupational class and household wealth.

**Conclusions:**

Disadvantageous childhood and adulthood socioeconomic circumstances were associated with lower long-term fruit and vegetable consumption. Socioeconomic circumstances should be considered in attempts to promote fruit and vegetable consumption, and people with disadvantageous circumstances need to be targeted in future interventions.

## Introduction

There is strong evidence on the importance of fruit and vegetable (F&V) consumption in the prevention of all major chronic diseases [1–3]. Adequate F&V consumption has been shown to be associated with a lower risk of obesity, cardiovascular disease, cancer and type 2 diabetes [4–6]. Improvement in dietary quality, which includes an increase in the proportion of F&Vs, could markedly reduce premature mortality globally [7]. A high proportion of F&Vs in the diet has been suggested to be beneficial also in terms of mental health and quality of life [8, 9].

Despite the fact that F&V intake is clearly one of the most important components and indicators of a health-promoting diet, recommended consumption levels are not reached in western countries [1]. In Finland, the most recent national survey showed that only 14% of men and 22% of women reach the recommended daily minimum consumption level, 500 g, of vegetables, fruit and berries [10].

Within a population, F&V consumption varies by sociodemographic characteristics. Differences have been found according to age and sex as well as living arrangements and environments [11–13]. Socioeconomic position has been a particularly strong determinant of F&V use. People in lower occupational classes and with lower education and incomes have been shown to purchase and consume F&Vs less frequently compared to those in higher positions [11, 12, 14]. Socioeconomic differences have also been shown to be more pronounced in F&V consumption than in the consumption of other foods [12]. Studies have, however, rarely examined, or compared, multiple socioeconomic indicators simultaneously, although this would be needed in order to evaluate better the importance of different aspects of socioeconomic circumstances in terms of public health policies and measures targeting dietary habits. There is some evidence showing that associations between socioeconomic circumstances and F&V consumption may differ according to the indicators used. In the Helsinki Health Study (HHS) cohort, income was shown to be a stronger determinant of F&V consumption than education [14]. Another limitation in previous studies has been the use of cross-sectional designs, and thus possible changes in F&V consumption have seldom been investigated.

This study examined long-term trajectories of F&V consumption among midlife and ageing people initially employed in the municipal sector in Finland. Multiple past and present socioeconomic circumstances – grouped into childhood circumstances (parental education, childhood economic circumstances), conventional socioeconomic circumstances (e.g. education) and material circumstances (e.g. wealth) – were examined as determinants of F&V consumption trajectories.

## Methods

### Data

We used survey data from four phases of the HHS among employees of the City of Helsinki, Finland. The phase 1 survey was conducted in 2000–2002 among employees reaching the age of 40, 45, 50, 55 and 60 years each year (*N*=8960, response rate 67%) [15]. The phase 2 follow-up survey was conducted in 2007 (*N*=7332, 83% responded), phase 3 in 2012 (*N*=6814, 79% responded) and phase 4 in 2017 (*N*=6832, 82% responded). The final number of participants who had full information on F&V consumption at all four survey phases was 5430 (83% women). The ethical aspects of the HHS have been approved by ethics committees at the Department of Public Health, University of Helsinki and the City of Helsinki health authorities.

### Measurement of fruit and vegetable consumption

All four survey phases included an identical 22-item food frequency questionnaire (FFQ). In the FFQ, participants were asked to estimate how often they had consumed the food items during the past 4 weeks. The items that were included in this study were ‘fruit and berries’ and ‘fresh vegetables, root vegetables, salads’. The response categories were: ‘not during the past 4 weeks’, ‘1–3 times a month’, ‘once a week’, ‘2–4 times a week’, ‘5–6 times a week’, ‘once a day’ and ‘two times or more daily’. For each response category, the average frequency per day was calculated and then multiplied by 28 days to produce the total number of consumption times per month: 0, 2, 4, 12, 22, 28 and 56, following our previous study [16].

### Indicators of socioeconomic circumstances

Childhood socioeconomic circumstances included parental education and childhood economic difficulties. Parental education was based on both mother’s and father’s educational level, of which the higher one was chosen and categorised into three levels: higher (matriculation or college examination or higher), intermediate (secondary school or vocational training) and basic (primary school or lower). Childhood economic difficulties were measured by asking whether the respondent’s childhood family had faced serious financial problems before the respondent was aged 16 years (yes/no).

Conventional adulthood socioeconomic circumstances included own education, occupational class and household income. Own education was divided into three levels: higher (university degree), intermediate (matriculation or college examination) and basic (secondary or vocational school). Occupational class included four hierarchical categories: administrative/managerial, professional/semiprofessional, non-manual employees and manual workers. The information was derived from the personnel register data (80%) and completed from survey data. Household income was based on the total typical monthly income. The monthly income was divided by household size and weighted according to the modified Organisation for Economic Co-operation and Development equivalence scale, which means that the respondent received the value of 1.0, other adults 0.5 and children 0.3. Four hierarchical income groups were formed with each of them consisting of approximately a quarter of the study population.

Material socioeconomic circumstances consisted of housing tenure and current economic difficulties. Housing tenure was dichotomised into owner-occupiers and renters. Current economic difficulties were measured with two questions from Pearlin’s list of chronic strains [17]: ‘How much difficulty do you have in meeting the payment of bills?’ and ‘How often do you have enough money to buy the food or clothing you or your family need?’. Five response alternatives indicated the level of difficulties: ‘very little’ to ‘very great’ for the first question, and ‘always’ to ‘never’ for the second question. A sum score was formed and categorised into no, occasional and frequent difficulties. Household wealth was measured with a question enquiring about the amount of money the respondent would have if all household assets were cashed and all debts paid off. Four hierarchical categories were formed: under €10,000; €10,000–99,999; €100,000–299,999; and €300,000 and over. Socioeconomic indicators were derived from the phase 1 survey except for wealth which was measured at phase 2.

### Covariates

Covariates included age, sex and retirement status. Participants reporting that they were on statutory or disability retirement at any of the follow-up survey phases were considered as having transitioned to retirement. The covariates were chosen on the basis of previous studies which have shown sociodemographic differences in F&V consumption [10, 11, 16].

### Statistical analyses

First, we calculated descriptive numbers and percentages for the socioeconomic indicators and covariates. Second, we identified F&V consumption trajectories using group-based trajectory modelling (GBTM). GBTM is an application of finite mixture modelling. It assumes that a population is composed of distinct subpopulations that follow a somewhat similar developmental pathway in the outcome of interest [18]. We used the following statistical criteria as the basis of choosing the number of optimal trajectories and their shapes: Bayesian information criteria, the average of posterior probabilities of group membership higher than 0.7, the odds of correct classification higher than 5.0 and the size of each trajectory group larger than 5% of participants [18]. Each participant was assigned to the trajectory for which they had the highest group membership probability. The model fit statistics are shown in Supplemental Table I.

Third, we fitted multinomial logistic regression models producing odds ratios (ORs) with 95% confidence intervals (CIs) to examine associations between the three groups of childhood and adulthood socioeconomic circumstances and trajectory group memberships. Base models adjusting for covariates, that is, age, sex and retirement status, were first fitted for each socioeconomic indicator (model 1). After that, the socioeconomic indicators were grouped into: (a) childhood circumstances (parental education, childhood economic difficulties); (b) adulthood conventional circumstances (own education, occupational class, household income); and (c) material (housing tenure, current economic difficulties, household wealth) socioeconomic circumstances, and models were fitted simultaneously to adjust for the circumstances in each of the three groups (model 2). For childhood circumstances, further models adjusting for the adulthood conventional (model 3) and material socioeconomic circumstances (model 4) were also fitted.

The analyses were performed using STATA version 15 with TRAJ command for identifying the trajectories, and SAS statistical software version 9.4 (SAS Institute Inc., Cary, NC, USA) for the regression models and descriptive analyses.

## Results

**Figure 1.**
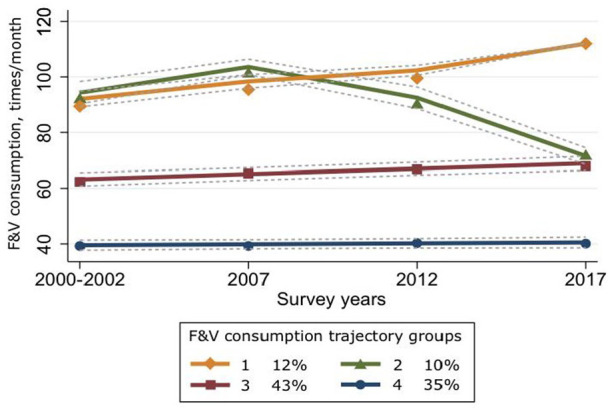
Fruit and vegetable (F&V) consumption trajectories and their prevalence (%), identified by group-based trajectory modelling (group means and fitted lines with 95% confidence intervals). Trajectory 1 ‘increasing high’, trajectory 2 ‘decreasing high’, trajectory 3 ‘stable moderate’ and trajectory 4 ‘stable low’.

The distributions of participants are presented in Table I. Four trajectories of F&V consumption were identified based on the model selection criteria of GBTM (Figure 1): increasing higher, decreasing higher, stable moderate and stable low. A major part of the participants was assigned to the two lowest trajectories: 35% of participants to the stable low and 43% of participants to the stable moderate trajectory. A fifth of the participants was assigned to one of the two higher trajectories: 10% to the decreasing higher and 12% to the increasing higher trajectory. The increasing higher trajectory gradually increased over the survey years, whereas the decreasing higher trajectory reached the highest point at phase 2, 2007, and then gradually decreased and ended very near to the stable moderate trajectory at the end of the follow-up.

**Table I.** Distribution of participants (*N*, %) by covariates and socioeconomic indicators.

	*N*	%
All	5430	100
Sociodemographic covariates		
Sex		
Women	4484	83
Men	946	17
Age at phase 1 (years)		
40	952	18
45	1114	21
50	1203	22
55	1473	27
60	688	13
Retirement status		
No	1943	36
Yes	3487	64
Childhood socioeconomic circumstances		
Parental education		
Higher	1205	22
Intermediate	1372	25
Basic	2810	52
Childhood economic difficulties		
No	4035	81
Yes	972	19
Conventional socioeconomic circumstances		
Own education		
Higher	1527	28
Intermediate	1738	32
Basic	2121	39
Occupational class		
Administrative and managerial	1711	32
Professional and semiprofessional	1119	21
Routine non-manual	1767	33
Manual workers	752	14
Household income		
Highest	1451	27
2nd	1250	24
3rd	1337	25
Lowest	1266	24
Material socioeconomic circumstances		
Housing tenure		
Owner-occupier	3781	70
Renter	1615	30
Current economic difficulties		
No	2952	55
Occasional	1916	36
Frequent	514	9
Wealth		
Highest	1752	35
2nd	1710	34
3rd	897	18
Lowest	635	13

Associations with F&V consumption trajectories were found in all three groups of socioeconomic circumstances (Tables II and III). In general, the ORs increased when moving from higher F&V trajectories towards lower trajectories. Among childhood circumstances (Table II), low parental education was associated most clearly with the stable low trajectory (model 1, OR 1.57, CI 1.25–1.97) but also with the stable moderate and decreasing higher trajectories. Childhood economic difficulties showed an association with the stable low trajectory (OR 1.66, CI 1.29–2.14). Mutual adjustment for the childhood circumstances (model 2) had only very slight effects on the ORs. Adjusting for adulthood circumstances in models 3 and 4 attenuated the initially observed associations, but childhood economic difficulties remained associated with the stable low trajectory after adjustment for conventional (OR 1.36, CI 1.05–1.78) and material (OR 1.35, CI 1.01–1.76) socioeconomic circumstances.

**Table II.** Associations of childhood socioeconomic circumstances with fruit and vegetable (F&V) consumption trajectories, odds ratios with 95% confidence intervals from multinomial logistic regression models.

	Trajectories of F&V consumption			
	Increasing	Decreasing	Stable	Stable
	Higher	Higher	Moderate	Low
Model 1a adjusted for covariates^a^				
Parental education (ref. high)				
Intermediate	1.00	1.42 (1.01–1.98)	1.28 (1.00–1.63)	1.38 (1.06–1.79)
Low	1.00	1.34 (1.00–1.80)	1.26 (1.01–1.56)	1.57 (1.25–1–97)
Model 1b adjusted for covariates^a^				
Childhood economic difficulties (ref. no)				
Yes	1.00	1.33 (0.97–1.82)	1.23 (0.96–1.58)	1.66 (1.29–2.14)
Model 2 adjusted mutually^b^				
Parental education (ref. high)				
Intermediate	1.00	1.40 (1.00–1.97)	1.25 (0.97–1.61)	1.33 (1.01–1.74)
Low	1.00	1.30 (0.95–1.77)	1.26 (1.00–1.57)	1.54 (1.21–1.95)
Childhood economic difficulties (ref. no)				
Yes	1.00	1.29 (0.94–1.77)	1.20 (0.93–1.54)	1.58 (1.22–2.04)
Model 3 adjusted for adulthood conventional socioeconomic circumstances^c^				
Parental education (ref. high)				
Intermediate	1.00	1.37 (0.95–1.96)	1.21 (0.93–1.58)	1.03 (0.77–1.36)
Low	1.00	1.26 (0.90–1.76)	1.18 (0.92–1.51)	1.02 (0.79–1.33)
Childhood economic difficulties (ref. no)				
Yes	1.00	1.26 (0.91–1.74)	1.13 (0.87–1.45)	1.36 (1.05–1.78)
Model 4 adjusted for adulthood material circumstances^d^				
Parental education (ref. high)				
Intermediate	1.00	1.41 (0.98–2.01)	1.26 (0.97–1.64)	1.19 (0.90–1.58)
Low	1.00	1.35 (0.98–1.87)	1.25 (0.98–1.58)	1.28 (0.99–1.64)
Childhood economic difficulties (ref. no)				
Yes	1.00	1.32 (0.94–1.84)	1.16 (0.89–1.52)	1.35 (1.01–1.76)

a Covariates: age, sex, retirement status.

b Covariates plus parental education, childhood economic difficulties.

c Covariates plus parental education, childhood economic difficulties, own education, occupational class, household income.

d Covariates plus parental education, childhood economic difficulties, housing tenure, current economic difficulties, household wealth.

**Table III.** Associations of adulthood conventional and material socioeconomic circumstances with fruit and vegetable (F&V) consumption trajectories, odds ratios with 95% confidence intervals from multinomial logistic regression models.

	Trajectories of F&V consumption			
	Increasing	Decreasing	Stable	Stable
	Higher	Higher	Moderate	Low
Model 1 adjusted for covariates^a^				
Own education (ref. high)				
Intermediate	1.00	1.20 (0.90–1.61)	1.10 (0.88–1.36)	1.18 (0.94–1.49)
Low	1.00	1.28 (0.95–1.72)	1.38 (1.10–1.72)	2.80 (2.22–3.54)
Occupational class (ref. highest)				
2nd	1.00	1.13 (0.83–1.55)	0.95 (0.75–1.20)	2.60 (2.15–3.15)
3rd	1.00	1.28 (0.95–1.71)	1.41 (1.13–1.75)	2.23 (1.76–2.82)
Lowest	1.00	1.30 (0.83–2.05)	1.66 (1.18–2.34)	3.96 (2.81–5.59)
Household income (ref. highest)				
2nd	1.00	0.93 (0.68–1.28)	1.06 (0.83–1.34)	1.44 (1.11–1.86)
3rd	1.00	0.96 (0.69–1.33)	1.30 (1.02–1.66)	2.06 (1.59–2.67)
Lowest	1.00	1.20 (0.86–1.68)	1.42 (1.10–1.84)	2.67 (2.03–3.49)
Model 2 mutually adjusted^b^				
Own education (ref. high)				
Intermediate	1.00	1.16 (0.79–1.71)	1.05 (0.78–1.39)	1.02 (0.75–1.39)
Low	1.00	1.14 (0.70–1.86)	0.98 (0.68–1.42)	1.50 (1.01–2.21)
Occupational class (ref. highest)				
2nd	1.00	1.03 (0.69–1.54)	0.88 (0.65–1.20)	0.85 (0.61–1.18)
3rd	1.00	1.11 (0.69–1.77)	1.29 (0.91–1.84)	1.41 (0.97–2.06)
Lowest	1.00	1.13 (0.61–2.11)	1.54 (0.97–2.45)	2.21 (1.37–3.57)
Household income (ref. highest)				
2nd	1.00	0.94 (0.68–1.30)	1.02 (0.80–1.30)	1.27 (0.98–1.65)
3rd	1.00	0.95 (0.68–1.35)	1.24 (0.96–1.61)	1.66 (1.26–2.18)
Lowest	1.00	1.16 (0.81–1.67)	1.24 (0.94–1.64)	1.81 (1.35–2.43)
Model 3 adjusted for covariates^a^				
Housing tenure (ref. owner-occupier)				
Renter	1.00	1.09 (0.83–1.44)	1.32 (1.07–1.62)	2.08 (1.68–2.57)
Current economic difficulties (ref. no)				
Occasional	1.00	0.97 (0.75–1.26)	1.15 (0.95–1.40)	1.92 (1.57–2.35)
Frequent	1.00	0.89 (0.56–1.41)	1.11 (0.80–1.55)	2.48 (1.77–3.45)
Household wealth (ref. highest)				
2nd	1.00	0.90 (0.68–1.19)	1.30 (1.05–1.60)	1.68 (1.34–2.10)
3rd	1.00	1.03 (0.72–1.46)	1.28 (0.98–1.67)	2.43 (1.84–3.21)
Lowest	1.00	1.36 (0.87–2.13)	1.86 (1.32–2.64)	4.53 (3.18–6.45)
Model 4 mutually adjusted^c^				
Housing tenure (ref. owner-occupier)				
Renter	1.00	0.94 (0.65–1.38)	1.05 (0.79–1.39)	1.03 (0.77–1.38)
Current economic difficulties (ref. no)				
Occasional	1.00	0.97 (0.73–1.28)	0.98 (0.80–1.20)	1.44 (1.16–1.79)
Frequent	1.00	0.87 (0.53–1.44)	1.01 (0.70–1.45)	1.77 (1.23–2.56)
Household wealth (ref. highest)				
2nd	1.00	0.90 (0.67–1.20)	1.30 (1.05–1.60)	1.57 (1.24–1.98)
3rd	1.00	1.09 (0.72–1.65)	1.25 (0.91–1.71)	2.11 (1.53–2.93)
Lowest	1.00	1.45 (0.83–2.56)	1.76 (1.14–2.70)	3.53 (2.28–5.48)

a Covariates: age, sex, retirement status.

b Covariates plus own education, occupational class, household income.

c Covariates plus housing tenure, current economic difficulties, household wealth.

Among conventional socioeconomic circumstances (Table III, models 1 and 2), the strongest association with the F&V trajectories was found for occupational class (model 1, OR range 2.60, CI 2.15–3.15 to 3.96, CI 2.81–5.59). Low own education was also associated with the stable low (OR 2.80, CI 2.22–3.54) and stable moderate (OR 1.38, CI 1.10–1.72) trajectories. For household income, all groups lower than the highest were found to be associated with the stable low trajectory (OR range 1.44, CI 1.11–1.86 to 2.67, CI 2.03–3.49). The lowest income group was also associated with the stable moderate trajectory. Mutual adjustment for all conventional socioeconomic circumstances in model 2 attenuated the ORs with the strongest association remaining for occupational class.

Of material socioeconomic circumstances (Table III, models 3 and 4) and overall of all socioeconomic circumstances, the strongest associations were observed for household wealth (OR range 1.68, CI 1.34–2.10 to 4.53, CI 3.18–6.45). Living in rented housing and, especially, reporting current economic difficulties (occasional OR 1.92, CI 1.57–2.35; frequent OR 2.48, CI 1.77–3.45) were associated with belonging to the stable low F&V consumption trajectory. Mutual adjustment for all material socioeconomic circumstances in model 4 attenuated the observed associations but they remained statistically significant for current economic difficulties and wealth.

## Discussion

This study examined long-term trajectories of F&V consumption and the associations of multiple past and present socioeconomic circumstances with the trajectories among midlife and aging employees of the City of Helsinki, Finland. The main results of the study were that four distinct trajectories of F&V consumption could be identified, and disadvantageous socioeconomic circumstances from all groups, that is, childhood circumstances, conventional socioeconomic circumstances and material socioeconomic circumstances, were associated with belonging to trajectories of lower consumption. Strongest associations with the trajectories were found for wealth and occupational class.

The four trajectories of F&V consumption were stable lowest, stable moderate, decreasing higher and increasing higher trajectory. The majority of the participants, 78%, belonged to the two lowest trajectories which remained stable throughout the whole follow-up. This is in line with the Finnish national figures among the general population, which show that a low level of F&V consumption is common among the population and the majority falls behind from the recommended daily consumption level [10]. International observations have shown a similar situation [1]. Regarding the two higher trajectories, the stability differed from the lower trajectories, as there were slight changes over time. These changes, emerging mostly from the 2007 time point onwards, could in these age groups be related to transition to retirement. In our earlier study concerning the associations of statutory retirement with food habits, we found vegetable consumption to decrease among women and fruit consumption to increase among men after retiring [16]. In the present study, however, adjusting for retirement status throughout the follow-up as a covariate did not affect the associations observed between socioeconomic circumstances and the F&V trajectories. Overall, the long-term trajectories show longitudinal differences which cannot be captured with cross-sectional analyses: lower F&V consumption was more persistent over time, and the changes in F&V consumption took place among participants with higher consumption trajectories.

Childhood disadvantageous circumstances, that is, low parental education and economic difficulties, were in the present study clearly associated with belonging to the two lowest trajectories of F&V consumption. This result is in line with previous studies, which have examined single indicators of childhood socioeconomic position and found that parental education or occupational class is associated with adulthood, also later adulthood, F&V consumption [19–21]. Dietary habits and preferences have been suggested to be strongly formed in the early life-course, being influenced by the family environment, and therefore also affected by the possible social and economic adversity in the family [22, 23]. Dietary patterns have been found to persist from childhood to adolescence and adulthood at least to some degree [24]. Thus, in general, childhood socioeconomic circumstances could possibly be equally important contributors to adulthood F&V consumption as adulthood circumstances [19]. To test this, we modelled the childhood circumstances also adjusting simultaneously for adulthood circumstances. Adjusting for adulthood conventional and material socioeconomic circumstances attenuated the associations to a large part, with only one association remaining after adjusting for all adulthood circumstances. This could refer to adulthood circumstances potentially being more important in contributing to the eating habits than childhood circumstances. However, it should be noted that the consumption of F&Vs in childhood among the participants is not known, which could affect the associations.

The conventional measures of socioeconomic circumstances, that is, own education, occupational class and household income, showed independent associations with F&V consumption trajectories (Table III). These were, however, somewhat affected by the simultaneous adjustments, and associations that remained clearest were those found for occupational class and income. For education, the lowest level remained associated with the lowest F&V trajectory (Table III). In previous cross-sectional studies on our HHS data, occupational class was shown to mediate the association between education and healthy food habits including daily F&V use, and household income was found to be associated with fresh F&V consumption at all education levels [14, 25]. It is possible that although all of the examined conventional socioeconomic circumstances showed some associations in this study, occupational class and especially income could be more strongly associated with F&V consumption as they better reflect the actual economic resources that provide access to higher cost foods [26].

In addition to income, further measures of material circumstances, that is, housing tenure, current economic difficulties and household wealth, were clearly associated with F&V consumption trajectories. Less wealthy participants were especially more likely to have a low trajectory of F&V consumption. The measures of material circumstances can be seen to differ from the conventional socioeconomic circumstances somewhat in what kind of circumstances and aspects of the socioeconomic position they implicate [27]. Wealth and economic difficulties, in addition to income, indicate the financial situation which essentially determines whether the individual or a family has the concrete daily life economic resources to afford a certain dietary quality [26]. Wealth could be considered as a more long-term measure of material resources and prosperity compared to monthly or yearly income, reflecting economic stability and security perhaps accumulating all the way from the early life-course. Economic difficulties can take place regardless of the level of income, as they could originate from consumption habits or indebtedness and various life events and situations. Therefore, although the differences in income are relatively small in Finland and especially among the municipal sector employees, differences in the financial situation and available material resources could be magnified through taking wealth, and also economic difficulties, into account in addition to disposable income. It is notable that our results in this study showed differences especially by wealth among the employee population in which the poorest and most disadvantaged part of the population are not included.

In this study, material resources in general showed clear associations with F&V consumption trajectories. This highlights the possibility, which has also been suggested in previous studies, that through policies targeting the financial affordability of F&Vs in comparison to other foods, socioeconomic inequality in adherence to healthy dietary habits could be reduced. However, as all other socioeconomic indicators that were studied also showed associations, multiple aspects of socioeconomic circumstances clearly need to be considered when planning policies, interventions and further studies. Overall, reducing socioeconomic differences in dietary habits could further contribute to the reduction of the prevailing socioeconomic health differences and inequalities in society.

### Methodological considerations

This prospective study was based on repeated survey data from four follow-up phases which was a major strength. Each survey phase included an identical FFQ to measure food consumption, which allowed us to examine changes in F&V consumption over time. Previous studies have shown that short FFQs are suitable for measuring population or group-level changes in foods that are frequently consumed [28, 29]. The prospective data allowed for utilising a sophisticated method of analysis, GBTM, which has rarely been used for examining long-term food consumption development. In general, the results are likely to be, with caution, generalisable to aging employees in the municipal sector in Finland, and possibly elsewhere in the public sector. The HHS population can be considered as representative of the target population, the employees of the City of Helsinki. Response rates have been at least satisfactory in each phase, and non-participation analyses have suggested that the results are not seriously biased [15, 30]. Older employees and those with a higher occupational class and income are slightly overrepresented in the sample, compared to the target population [30].

The limitations include the characteristics of the survey data, which consisted of midlife and aging municipal employees. The majority of the participants were women which reflects the situation within the Finnish municipal sector in general but causes restrictions to generalisations and the statistical power among men. Limitations also apply to the measures that were used. The FFQ was not validated and did not include portions or portion sizes and consumption quantities could not be calculated. However, the contribution of portion size questions to the variance in food intake in FFQs has been shown to be negligible [31]. Also, the study period was long, from 2000 to 2017, and unmeasured changes to any direction could take place in between the four survey phases which were set 5 to 7 years apart.

## Conclusions

This study among midlife and aging people found trajectories showing F&V consumption to change relatively little over time and lower trajectories to be more common. Disadvantageous past and present socioeconomic circumstances, measured with multiple indicators, were associated with lower-level long-term F&V consumption trajectories. Different aspects of socioeconomic circumstances throughout the life-course should be considered in attempts to promote F&V consumption, and especially people with disadvantageous socioeconomic circumstances need to be targeted in future interventions.

## Supplemental Material

sj-docx-1-sjp-10.1177_14034948221094430 – Supplemental material for Multiple socioeconomic circumstances and trajectories of fruit and vegetable consumption: the Helsinki Health Study Supplemental material, sj-docx-1-sjp-10.1177_14034948221094430 for Multiple socioeconomic circumstances and trajectories of fruit and vegetable consumption: the Helsinki Health Study by Elina Mauramo, Jatta Salmela, Leonie H. Bogl, Tea Lallukka and Noora Kanerva in Scandinavian Journal of Public Health
