# Supplementary material for: Multiple socioeconomic circumstances and trajectories of fruit and vegetable consumption: the Helsinki Health Study
Source: Scand J Public Health. 2022 May 10;51(8):1144–52. doi: 10.1177/14034948221094430 (PMC11977821; doi:10.1177/14034948221094430)
Supplement: sj-docx-1-sjp-10.1177_14034948221094430 – Supplemental material for Multiple socioeconomic circumstances and trajectories of fruit and vegetable consumption: the Helsinki Health Study [file sj-docx-1-sjp-10.1177_14034948221094430.docx]

Supplementary Table S1. Model selection of optimal number of trajectory groups and shapes. Bayesian information criterion (BIC) and the group sizes shown, optimal model bolded.

| Number  of groups | Trajectory shapes^a^ | BIC^b^  (*n* = 21,720) | BIC^c^  (*n* = 5,430) | Group 1 (%) | Group 2 (%) | Group 3 (%) | Group 4 (%) |
| --- | --- | --- | --- | --- | --- | --- | --- |
| 1 | 3 | -94,798 | -94,794 | 100.0 |  |  |  |
| 2 | 3 3 | -91,811 | -91,804 | 68.7 | 31.3 |  |  |
| 3 | 3 3 3 | -91,162 | -91,152 | 47.3 | 40.3 | 12.4 |  |
| 4 | 3 3 3 3 | -90,862 | 90,848 | 35.8 | 42.9 | 9.7 | 11.6 |
| 4 | 2 3 3 3 | -90,858 | -90,844 | 35.8 | 42.9 | 9.7 | 11.6 |
| 4 | 2 2 3 3 | -90,853 | -90,840 | 35.8 | 42.9 | 9.7 | 11.6 |
| 4 | 2 2 2 3 | -90,852 | -90,84 | 34.9 | 43.3 | 10.0 | 11.8 |
| 4 | 2 2 2 2 | -90,874 | -90,863 | 48.4 | 36.6 | 7.0 | 8.0 |
| 4 | 1 2 2 3 | -90,847 | -90,836 | 34.9 | 43.3 | 10.0 | 11.8 |
| 4 | 1 1 2 3 | -90,846 | -90,835 | 34.6 | 43.3 | 10.4 | 11.8 |
| 4 | 1 1 1 3 | -90,969 | -90960 | 26.2 | 45.7 | 22.4 | 5.7 |
| **4** | **1 1 3 3** | **-90,845** | **-90,834** | **35.8** | **42.7** | **9.9** | **11.6** |
| 4 | 1 3 3 3 | -90,853 | -90,840 | 35.8 | 42.9 | 9.7 | 11.6 |
| 4 | 1 1 2 2 | -90,887 | -90,877 | 37.4 | 42.2 | 9.7 | 10.7 |
| 4 | 1 1 1 1 | -90,912 | -90,904 | 43.8 | 39.8 | 7.3 | 9.1 |

^a^ Trajectory shapes: 0 = intercept, 1 = linear, 2 = quadratic, 3 = cubic.

^b^ Bayesian information criterion (BIC) in longitudinal level.

^c^ Bayesian information criterion (BIC) in subject level.
